# Exosomes derived from miR-146a-overexpressing fibroblast-like synoviocytes in cartilage degradation and macrophage M1 polarization: a novel protective agent for osteoarthritis?

**DOI:** 10.3389/fimmu.2024.1361606

**Published:** 2024-05-23

**Authors:** Huan Wang, Yue Zhang, Chengfei Zhang, Yan Zhao, Jun Shu, Xuezhang Tang

**Affiliations:** ^1^ Department of Traditional Chinese Medicine Massage, China-Japan Friendship Hospital, Beijing, China; ^2^ School of Life Sciences, Beijing University of Chinese Medicine, Beijing, China; ^3^ Department of Endocrinology, Dongfang Hospital, Beijing University of Chinese Medicine, Beijing, China; ^4^ Department of Subhealth, Dongfang Hospital, Beijing University of Chinese Medicine, Beijing, China; ^5^ Institute of Clinical Research, China-Japan Friendship Hospital, Beijing, China

**Keywords:** osteoarthritis, miR-146a, exosome, cartilage degradation, macrophage polarization

## Abstract

**Introduction:**

Pathological changes in the articular cartilage (AC) and synovium are major manifestations of osteoarthritis (OA) and are strongly associated with pain and functional limitations. Exosome-derived microRNAs (miRNAs) are crucial regulatory factors in intercellular communication and can influence the progression of OA by participating in the degradation of chondrocytes and the phenotypic transformation in the polarization of synovial macrophages. However, the specific relationships and pathways of action of exosomal miRNAs in the pathological progression of OA in both cartilage and synovium remain unclear.

**Methods:**

This study evaluates the effects of fibroblast-like synoviocyte (FLS)-derived exosomes (FLS-Exos), influenced by miR-146a, on AC degradation and synovial macrophage polarization. We investigated the targeted relationship between miR-146a and TRAF6, both *in vivo* and *in vitro*, along with the involvement of the NF-κB signaling pathway.

**Results:**

The expression of miR-146a in the synovial exosomes of OA rats was significantly higher than in healthy rats. *In vitro*, the upregulation of miR-146a reduced chondrocyte apoptosis, whereas its downregulation had the opposite effect. *In vivo*, exosomes derived from miR-146a-overexpressing FLSs (miR-146a-FLS-Exos) reduced AC injury and chondrocyte apoptosis in OA. Furthermore, synovial proliferation was reduced, and the polarization of synovial macrophages shifted from M1 to M2. Mechanistically, the expression of TRAF6 was inhibited by targeting miR-146a, thereby modulating the Toll-like receptor 4/TRAF6/NF-κB pathway in the innate immune response.

**Discussion:**

These findings suggest that miR-146a, mediated through FLS-Exos, may alleviate OA progression by modulating cartilage degradation and macrophage polarization, implicating the NF-κB pathway in the innate immune response. These insights highlight the therapeutic potential of miR-146a as a protective agent in OA, underscoring the importance of exosomal miRNAs in the pathogenesis and potential treatment of the disease.

## 1 Introduction

Osteoarthritis (OA) is the most common form of arthritis, affecting more than 30% of individuals aged over 65 years (1–3). The incidence of the disease mirrors the trends of an aging population and a surge in risk factors like obesity and insufficient physical activity that predispose individuals to joint damage (4). Characterized by cartilage deterioration and synovial inflammation, OA leads to pain and reduced mobility, thereby escalating healthcare expenditure (5, 6). Current treatments primarily manage symptoms rather than halt or reverse the underlying joint damage, highlighting the urgent need for more effective therapeutic strategies (7).

At the heart of OA’s pathophysiology is macrophage polarization, a process vital for tissue equilibrium and disease outcome. Macrophages can polarize into pro-inflammatory M1 or anti-inflammatory M2 phenotypes, influenced by various stimuli. M1 macrophages release inflammatory cytokines that contribute to cartilage damage and synovial inflammation, whereas M2 macrophages promote inflammation resolution and tissue repair (8).

The TLR4/TRAF6/NF-kB pathway is central to macrophage polarization. Activation of TLR4 by PAMPs and DAMPs initiates a signaling cascade involving TRAF6 and leads to NF-kB translocation into the nucleus, where it stimulates the transcription of pro-inflammatory genes. This pathway is vital for M1 macrophage activation and is a promising target for OA interventions, as modulating this pathway may shift macrophage polarization towards an M2 phenotype, offering therapeutic benefits. Moreover, the interplay between macrophages and other joint tissue cells, mediated by signaling molecules such as microRNAs (miRNAs), is emerging as a crucial factor in OA pathogenesis (9).

In recent years, with more studies on the cell microenvironment, intercellular communication has received increasing attention. Notably, exosomes, as nanoscale vesicles released by almost all types of cells, can wrap and deliver a large amount of genetic information, such as microRNAs (miRNAs), and change key processes, such as cell survival and differentiation, thus, affecting the progression of diseases (10). High levels of exosomes have been observed in synovial fluid. Fibroblast-like synoviocytes (FLSs), the most common cell type in synovial fluid, are the main cell type involved in exosome secretion. The bioactive substances released by exosomes secreted into the synovial fluid are important determinants of OA-related pathological manifestations, such as chondrocyte apoptosis and synovial macrophage phenotype changes (11–13). The miRNAs delivered by exosomes are strong, post-transcriptional regulators of OA, regulate gene expression by targeting specific mRNAs, participate in various signal transduction activities to determine cell fate, and are active performers in the regulatory barrier of the innate immune response against the invasion of various pathogens (14). Of these, miR-146a, the first miRNA found to regulate the immune system, is a biomarker of OA (15). Reportedly, miR-146a is substantially upregulated in OA and is associated with the clinicopathological features of patients as a potential regulator of joint health (16). However, there is little evidence to show how miR-146a, with the help of exosomes, which are natural nanoscale carriers, plays a role in joint pathology and the main articular structure cartilage and synovium of OA, and its specific mechanism. In this study, the roles of miR-146a and FLS-derived exosomes (FLS-Exos) in cartilage degradation and synovial macrophage polarization were investigated *in vivo* and *in vitro*, and the possible molecular mechanisms underlying these roles were analyzed. This study elucidates the role of exosomal miRNAs in OA and emphasizes that exosomes derived from miR-146a-overexpressing FLSs (miR-146a-FLS-Exos) may be novel therapeutic tools for the treatment of OA.

## 2 Materials and methods

### 2.1 Establishment of a knee OA model in rats

Forty, eight-week-old adult female Wistar rats (SLAC, Shanghai, China), SPF grade, weighing 220–240 g, were fed adaptively for 1 week, and an OA model was constructed using the modified Hulth method (17). The model group, consisting of 20 rats, was anesthetized by a peritoneal injection of 2% pentobarbital sodium solution (Sinopharm, Beijing, China) and fixed in the lateral position. The incision of the right knee joint was clipped and disinfected, and a longitudinal skin incision was made to cut the anterior cruciate and medial collateral ligaments and resected the medial meniscus to destroy the stability of the knee joint of the rat with minor damage, concentrate the stress, and induce OA. The control group, consisting of 20 rats, was not surgically treated. The model and control groups were fed routinely under the same conditions. After eight weeks, knee synovial fluid was collected from both groups, and exosomes were separated from the synovial fluid. All animal experiments were approved by the Ethics Committee for Animal Experiments of the China–Japan Friendship Hospital (No.180117) and extensive efforts were made to minimize the use and suffering of animals in accordance with the Guidelines for the Care and Use of Experimental Animals (18).

### 2.2 Isolation and identification of exosomes

Exosomes in the synovial fluid were isolated using exosome kits by following the manufacturer’s instructions. Exosomes in the cell supernatant were separated by differential centrifugation (Optima XE, Beckman, Fort Collins, CO, USA). Specifically, the collected cell supernatant samples were centrifuged at 4°C at 500 × *g* (10 min), and the supernatants were transferred to a sterile centrifuge tube. They were then centrifuged at 2,000 × *g* for 30 min, followed by 10,000 × *g* for 30 min. The supernatant was collected, supplemented with sterile phosphate-buffered saline (PBS, Corning, NY, USA), and centrifuged at 120,000 × *g* (1 h). The precipitates obtained after the removal of the supernatant were exosome particles, which were resuspended with sterile PBS and stored at –80°C. The collected exosome-like vesicles were characterized by transmission electron microscopy (TEM, Tecnai f20, Philips, Amsterdam, Netherlands). Particle size was analyzed using nanoparticle tracking analysis (NTA, NS300, NanoSight, Malvern, UK). The expression of the exosome surface-specific marker protein CD9, tumor susceptibility gene 101 (TSG101), and heat shock protein 70 (HSP70) was assessed using western blotting (WB).

### 2.3 Functional analysis and target gene verification of exosomal miR-146a

Real-time quantitative polymerase chain reaction (qPCR) was used to detect whether there were marked differences in the expression level of miR-146a in the exosome samples of OA and healthy rat synovial fluid to confirm whether miR-146a was an exosomal miRNA affecting pathological changes in OA. GO and KEGG were used for functional enrichment analysis of miR-146a, and the signaling pathways and targeted genes related to the progression of OA were screened. The TargetScan website was used to predict the targeting relationship and binding site information of miR-146a and its target genes, and double-luciferase reporter gene detection was used for verification. Double-luciferase reporter vectors of the target gene, TRAF6 at the binding site of miR-146a; namely, pGL3-TRAF6 wild-type (WT) and pGL3-TRAF6 mutant vectors, were constructed. The two vectors were co-transfected into HEK293 cells (Cell Bank of Chinese Academy of Sciences, Shanghai, China) with miR-146a and control plasmids. Subsequently, 24 h after transfection, the cells were lysed and the supernatant was collected as instructed by the dual luciferase reporting kit (Beyotime, Shanghai, China). A multifunctional enzyme marker with a chemiluminescence detection function was used to detect the activities of firefly and sea kidney luciferases, that ratio of which represented relative luciferase activity. The sequences (Generay Biotech, Shanghai, China) used for the double-luciferase assay were: pGL3-TRAF6-WT-3′ UTR, TTCTGAGCTGGGGTTTGTGCTGGC; pGL3- TRAF6-MUT-3′ UTR, TAGTGAGGTGCCGTTACAGGTGCC; miR-146a mimic, sense 5′- UGAGAACUGAAUUCCAUGGGUU-3′, antisense 5′-AACCCAUGGAAUUCAGUUCUCA-3′; miR-146a mimic NC, sense 5′- UUUGUACACAAAAGUACUG-3′, and antisense 5′-CAGUACUUUUGUGUAGUACAAA-4′.

### 2.4 Isolation, culture, and transfection of chondrocytes and FLSs

Primary articular chondrocytes and FLSs were separated from the knee joints of 20 OA rats using collagenase. After rinsing with PBS solution several times, the rat cartilage and synovial tissue samples were separated on an ultra-clean experimental table and broken into small particles with ophthalmic scissors as far as possible. Then, 5 mL of type II collagens (Sigma-Aldrich, St. Louis, MO, USA) was added and cultured in a 5% CO_2_ humidity incubator at 37°C for 6 h for cartilage and 4 h for synovium. The cell suspensions were collected using a 100-mesh cell filter. After centrifugation at 500 × *g* (10 min), the cells were inoculated into complete DMEM/F12 (Gibco, Carlsbad, CA, USA) containing 10% fetal bovine serum (Gibco) for culture and subculture. The cells from 3–5 generations were used for follow-up experiments. When the cell supernatant was used for subsequent exosome extraction, it was replaced with culture medium without exosomes. Transfection was performed when cells reached approximately 80% confluence. According to the manufacturer’s instructions, miR-146a mimics, inhibitors, or the corresponding negative controls (GenerayBiotech) were transfected into chondrocytes using Lipofectamine 2000 (Invitrogen, Carlsbad, CA, USA). After pretreating FLSs with the miR-146a mimic plasmid, miR-146a was overexpressed in the FLSs. Simultaneously, a corresponding negative control group was established, along with a blank group without any transfection treatment, to control the influence of miR-146a overexpression; exosome particles in the cell supernatants of the two groups were collected and named miR-146a-FLS-Exos and miR-146a-NC-FLS-Exos, respectively, and their identities were evaluated using TEM, NTA, and WB. The sequences of the oligonucleotides used were: miR-146a inhibitor, 5′-AACCCAUGGAAUUCAGUUCUCA-3′; miR-146a inhibitor NC, 5′- CAGUACUUUUGUGUAGUACAAA-5′.

### 2.5 Functional experiment of FLS-miR-146a-Exos *in vivo*

The rats were divided into the model, control, miR-146a-NC, and miR-146a groups with 20 rats in each. The miR-146a-NC and miR-146a groups of OA rats received injections of miR-146a-NC-FLS-Exos and miR-146a-FLS-Exos in the injured joints, respectively, for 4–8 weeks after OA modeling (100 μg/rat, twice a week). Simultaneously, the model (OA rats) and control groups (healthy rats) without exosome treatment were used to evaluate the function of miR-146a-FLS-Exos in knee injury in OA rats. Stimulation responses, such as local pain, gait change, joint range of motion, and degree of joint swelling, were closely observed. Behavioral scores were calculated according to the modified Lequesne MG index (19), and cartilage and synovial tissue samples were collected from rats in each group eight weeks after modeling.

### 2.6 TUNEL staining

Chondrocyte apoptosis was observed using a one-step TUNEL cell apoptosis assay kit (Beyotime) based on broken DNA labeled with terminal deoxynucleotidyl-transferase. First, the cartilage sections were hydrolyzed with protease K without DNase at room temperature for 15 min, then TUNEL staining solution was added and the mixture was incubated at 37°C for 60 min in the dark. After the tablet was sealed with anti-fluorescence and anti-quenching sealing solutions, positive TUNEL staining of chondrocytes in the visual field was observed under a fluorescence microscope.

### 2.7 Histological analysis

The cartilage and synovial tissue of the knee joints was carefully peeled and placed in a 4% paraformaldehyde fixation solution (Sigma-Aldrich, St. Louis, MO, USA) for 24 h. The cartilage underwent another 4 weeks of EDTA (Servicebio Tech., Wuhan, China) microwave-assisted decalcification, whereas the synovium did not. The samples were routinely dehydrated, made transparent with a gradient of ethanol and xylene, and embedded in paraffin. Cartilage and synovium sections with a thickness of 4 μm were prepared and heated at 60°C for 30 min. Xylene and gradient ethanol were used for routine dewaxing and rehydration, and sections were stained according to the manufacturer’s instructions. Hematoxylin and eosin (HE) staining (Servicebio Tech.) was used to observe the morphological characteristics of the AC and synovium, and fersin O-solid green staining (Servicebio Tech.) was used to observe changes in proteoglycan content in the cartilage matrix of each group. Finally, the sections were closed with neutral glue and observed and photographed under a light microscope.

### 2.8 Immunofluorescence co-localization analysis

The levels of the M1-polarized macrophage marker iNOS (Abcam, Cambridge, MA, USA) and the M2-polarized macrophage marker CD206 (Abcam) in the synovium were analyzed by immunofluorescence co-localization analysis. Synovial sections were placed in a repair box filled with EDTA antigen repair buffer using the microwave method for antigen repair. The sections were then incubated with 3% BSA (Sigma-Aldrich) for 30 min. The sections were incubated overnight at 4°C with iNOS and CD206 primary antibodies using F4/80. Cy3-goat anti-rabbit or 488-goat anti-mouse fluorescent secondary antibodies labeled with horseradish peroxidase (HRP, Abcam) were incubated at room temperature for 60 min and protected from light. Then, an anti-fluorescence quencher containing DAPI (Beyotime) was added and the tablet was sealed. Finally, the sections were placed under a fluorescence microscope (IX-70, Olympus, Tokyo, Japan) to observe the expression of positive cells and to collect images. The staining results were quantified according to the integrated optical density using ImageJ software (NIH, Bethesda, MA, USA).

### 2.9 Flow cytometry analysis

The collected chondrocytes were washed twice with Stain Buffer (BD Pharmingen, San Diego, CA, USA) at a rotating speed of 300 × *g* (5 min) and the chondrocyte samples were stained with Annexin V/FITC and PI (BD Pharmingen). After incubation in the dark for 15 min, flow cytometry was performed (LSRII, BD Biosciences, San Jose, CA, USA). FlowJo software (Tree Star Inc., Ashland, OR, USA) was used to analyze the data.

### 2.10 Western blotting analysis

Total protein was extracted from the exosomes, cells, and tissues using radioimmunoprecipitation lysis buffer containing the protease inhibitor, phenylmethanesulfonyl fluoride, on ice for 30 min. The solution was then centrifugally rotated at 12,000 rpm at 4°C for 15 min. The total protein content in the supernatant of each sample was determined using a bicinchoninic acid kit (Sigma-Aldrich). Proteins were separated using sodium dodecyl sulfate-polyacrylamide gel electrophoresis (Tris-HCL SDS-PAGE). The protein was transferred to polyvinylidene fluoride membrane and blocked with 5% skim milk for 2 h at 37°C. Then, antibodies against CD9 (1:1000), TSG101 (1:2000), HSP70 (1:1000), Toll-like receptor (TLR) 4 (1:1000), TRAF6 (1:1000), NF-κB p65 (1:2000), and phosphorylated NF-κB p65 (1:2000) (all from Abcam) were tested at 4°C overnight. Then, sheep anti-rabbit or sheep anti-mouse immunoglobulin G antibody (1: 10000, Jackson Immunoresearch, West Grove, PA, USA) labeled with HRP was incubated at 37°C for 2 h, then chemical luminescence (Millipore ECL system) was performed. Quantitative protein analysis was performed using ImageJ software (NIH).

### 2.11 qPCR

Total RNA was extracted from exosomes, cells, and tissues using RNAiso Plus (TRIzol, Thermo Fisher Scientific, Waltham, MA, USA), and absorbance at 260/280 nm was used to assess RNA purity and concentration. The PrimeScript™ II 1st Strand cDNA Synthesis Kit (TaKaRa, Tokyo, Japan) was used for reverse transcription, and the SYBR kit (Thermo Fisher Scientific) was used for fluorescence quantitative PCR amplification. The relative mRNA expression of the miR-146a, TLR4, TRAF6, and NF-κB genes was evaluated using the 2^−ΔΔ^ *^C^* ^T^ method, with glyceraldehyde-3-phosphate dehydrogenase (GAPDH) as the internal reference gene.

The primer sequences (Sangon Biotech, Shanghai, China) used in this study were: miR-146a, stem-loop 5′-GTCGTATCCAGTGCAGGGTCCGAGGTATTCGCACTGGATACGACAACCCA-3′, forward 5′-GCGCGCTGAGAACTGAATTCCA-3′; universal downstream primer for stem-loop method, 5′-GTGCAGGGTCCGAGGT-3′; U6, reverse transcription 5′-CGCTTCACGAATTTGCGTGTCAT-3′, forward 5′-GCTTCGGCAGCACATATACTAAAAT-3′, reverse 5′-CGCTTCACGAATTTGCGTGTCAT-3′; TLR4, forward 5′- AGACCTGTCCCTGAACCCTAT-3′, reverse 5′- CGATGGACTTCTAAACCAGCCA-3′; TRAF6, forward 5′- TTTGCTCTTATGGATTGTCCCC-3′, reverse 5′- CATTGATGCAGCACAGTTGTC-3′; NF-κB, forward 5′- AACAGAGAGGATTTCGTTTCCG-3′, reverse 5′- TTTGACCTGAGGGTAAGACTTCT-3′; GAPDH, forward 5′- TGACAACTTTGGTATCGTGGAAGG-3′, and reverse 5′- AGGCAGGGATGATGTTCTGGAGAG-3′.

### 2.12 Statistical analysis

All data are expressed as the mean ± standard error of the mean (SEM). SPSS 22.0 (Inc., Chicago, IL, USA) and GraphPad Prism 9.0 software (GraphPad Software, Inc., La Jolla, CA, USA) were used for data processing, and one-way analysis of variance was used to analyze the differences among the groups. Pairwise comparisons between the groups were performed using Student’s *t*-test. *P* < 0.05 was the significance threshold for this study.

## 3 Results

### 3.1 The synovial fluid contained exosomes

**Figure 1:**
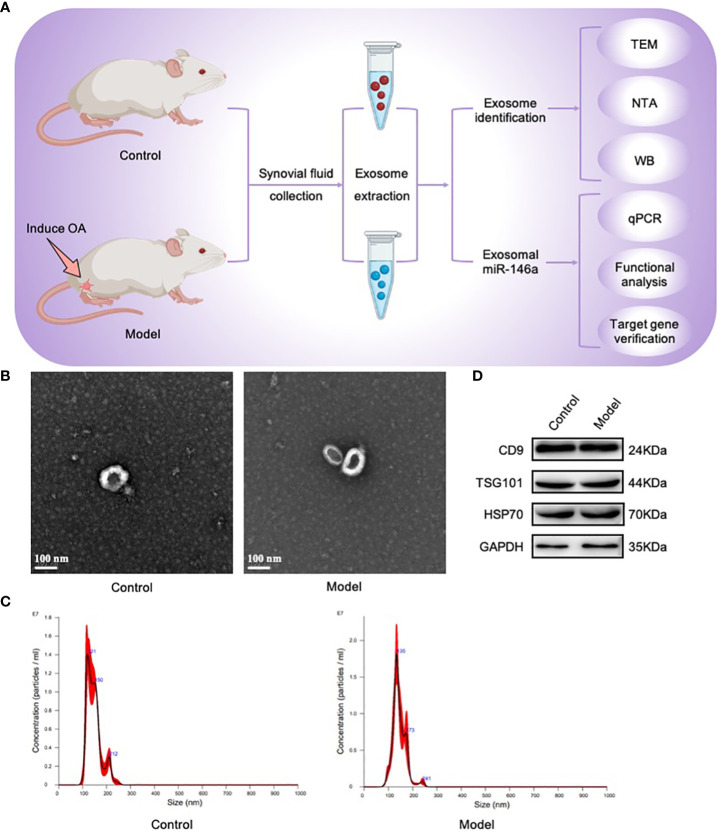
Exosomes were isolated and identified using TEM, NTA, and WB. **(A)** Joint synovial fluid samples were obtained from OA (model group) and healthy rats (control group). Exosomes were then collected for analysis. **(B)** The morphology of exosome-like vesicles was observed using TEM (×100000). **(C)** The exosome particle size was measured using NTA (nm). **(D)** WB detected the characteristic markers, CD9, TSG101, and HSP70, of exosomal vesicles.

First, by establishing the OA model required for this study, synovial fluid samples were collected from the OA model and healthy control groups; exosome particles in these samples were isolated and identified. Using TEM, nearly-hollow, spherical, saucer-shaped, or cup-shaped vesicles were observed in both groups under microscopic fields of view. NTA revealed that these exosomes were particles with peak particle sizes of 121 and 135 nm. WB analysis revealed that the extracted exosomes were positive for the exosome characteristic markers, CD9, TSG101, and HSP70. The above results showed that exosomes were present in the synovial fluid of the rat joints and were successfully collected (**Figure 1**).

### 3.2 Expression of miR-146a in the exosomes of synovial fluid was altered by OA

**Figure 2:**
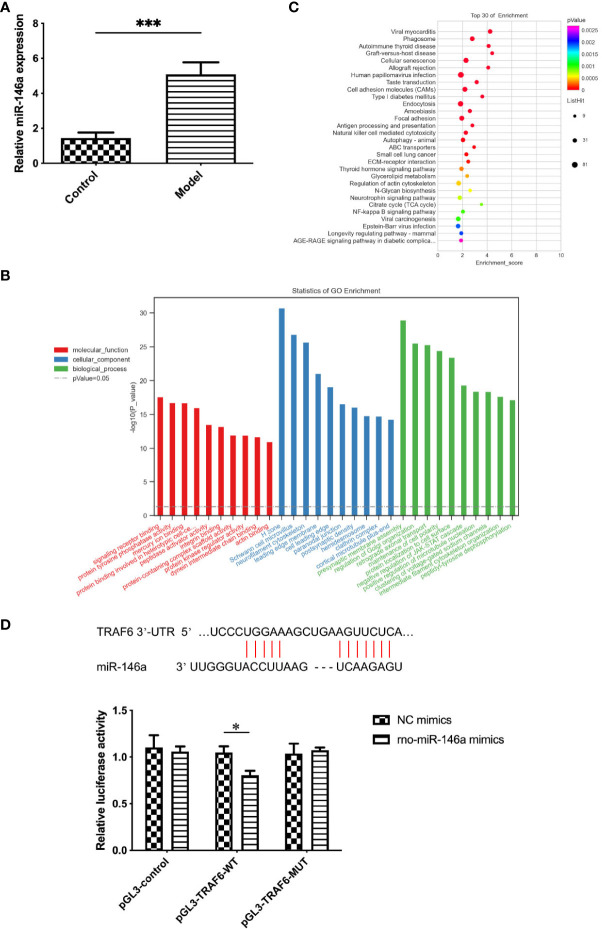
Functional enrichment analysis of exosomes and exosomal miR-146a in rat synovial fluid. **(A)** qPCR was used to detect the expression level of miR-146a in exosome samples of the synovial fluid. **(B, C)** GO and KEGG functional enrichment analyses were conducted. **(D)** Binding sites of the miR-146a targeting sequence in the TRAF6 3’ UTR. Luciferase reporter experiments were performed in HEK293 cells by transfecting miR-146a mimic, NC and WT, or MUT TRAF6 3’UTR luciferase reporter plasmids. Data are expressed as the mean ± SEM (n = 3). *** *p* < 0.0001, * *p* < 0.05.

Although the exosomes collected from the synovial fluid of OA and healthy rats were similar in morphology, size, concentration, and characteristics, significant differences were found in the expression level of miR-146a in the exosome samples of the two groups using qPCR. The expression level of miR-146a in the synovial fluid exosomes of rats in the model group was substantially higher than that in the control group. Additionally, miR-146a was speculated to be an exosomal miRNA that affects pathological changes in OA (**Figure 2A**).

### 3.3 miR-146a is closely related to the TLR4/TRAF6/NF-κB pathway in the innate immune response

Considering the possible effect of the differential expression of miR-146a on joint physiology and pathology in OA and healthy rats, GO and KEGG were used to conduct functional enrichment analysis of miR-146a. The miR-146a was found to play a role in signal receptor binding, protein kinase regulator binding, cell aging, and damage repair in three functional categories: molecular functions, cellular components, and biological processes. Although the NF-κB pathway was not the most prominent finding in our GO and KEGG analyses, its inclusion indicated its potential importance in our study. Some literature has reported the critical role of this pathway in innate immune mechanisms and its interactions with miR-146a. For example, the study by Wang et al. (20) revealed that miR-146a mimics can inhibit the expression of proteins related to the TLR4/TRAF6/NF-κB signaling pathway. Our previous research analyzed the NF-κB-related innate immune response pathways during the progression of OA, particularly their role in regulating synovial inflammation, which further supports the importance of this pathway in OA (21). These findings strengthened our understanding of the central role of the NF-κB pathway in the pathogenesis of OA and its potential impact on prevention, diagnosis, and treatment strategies. The innate immune response and NF-κB pathway are closely related to miR-146a, and TRAF6 is the target gene of miR-146a (**Figures 2B, C**). Furthermore, the targeting relationship between miR-146a and TRAF6 was confirmed using the TargetScan database and double luciferase assay. Since TLR4 is an important pattern recognition receptor associated with NF-κB and TRAF6 transduction pathways in the innate immune system, in this study, miR-146a, associated with the TLR4/TRAF6/NF-κB pathway in the innate immune response, was selected as the main object of the mechanism study (**Figure 2D**).

### 3.4 miR-146a inhibits chondrocyte apoptosis *in vitro*

**Figure 3:**
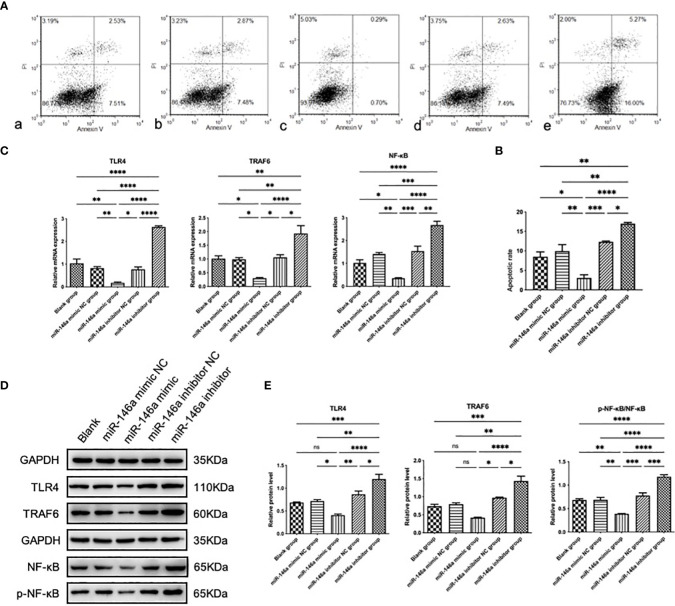
The effect and mechanism of miR-146a on chondrocyte apoptosis *in vitro*. **(A, B)** The apoptosis rate of chondrocytes was determined using FCM. Detection was performed 24 h after transfection with the miR-146a mimic or inhibitor. **(C)** Relative mRNA expression levels of TLR4, TRAF6, and NF-κB were detected using qPCR. **(D, E)** The protein levels of TLR4, TRAF6, p-NF-κB, and NF-κB were detected using WB. Data are expressed as the mean ± SEM (n = 3). **** *p* < 0.00001, *** *p* < 0.0001, ** *p* < 0.01, * *p* < 0.05.

Chondrocyte apoptosis is the primary pathological manifestation of OA. To further investigate the effect of miR-146a on chondrocyte growth and apoptosis, the effect of miR-146a on chondrocyte apoptosis was evaluated using flow cytometry with Annexin V-FITC/PI double staining. Rat articular chondrocytes were transfected and cultured using the collagenase separation method. The Annexin V-FITC/PI double-labeled parameter graph, combined with statistical analysis results, showed that the total apoptosis rate of chondrocytes transfected with the miR-146a mimic was significantly lower than that of chondrocytes transfected with the miR-146a mimic negative control. In contrast, the total apoptosis rate in chondrocytes transfected with the miR-146a inhibitor was significantly higher than that in chondrocytes transfected with the miR-146a inhibitor-negative control. No significant difference was observed in the total apoptosis rate of chondrocytes between the miR-146a-simulated and -inhibited negative controls and the blank group without intervention. After comparison, it was found that the changing trend in the early and late apoptosis rates of chondrocytes in each group was consistent with that of the total apoptosis rate. These results indicate that miR-146a can alleviate chondrocyte apoptosis to a certain extent (**Figures 3A, B**).

### 3.5 miR-146a inhibits the TLR4/TRAF6/NF-κB pathway *in vitro*

To the effect of miR-146a on the TLR4/TRAF6/NF-κB pathway, the protein expression levels of TLR4, TRAF6, phosphorylated NF-κB (p-NF-κB) and NF-κB were analyzed using WB. The ratio of p-NF-κB to total NF-κB was used to represent the activity of NF-κB. The expression levels of the above genes in chondrocytes transfected with the miR-146a mimic were significantly inhibited compared to those in the negative control group, whereas the expression levels of the above proteins were significantly increased after transfection with the miR-146a inhibitor. No significant differences in the TLR4, TRAF6, p-NF-κB/NF-κB protein levels between the negative control and blank groups without intervention was observed. The expression levels of the above mRNA were detected by qPCR, and the results were consistent with WB analysis. It was confirmed that miR-146a inhibits the TLR4/TRAF6/NF-κB pathway (**Figures 3C–E**).

### 3.6 miR-146a-FLS-Exos improves the behavioral scores of OA rats

**Figure 4:**
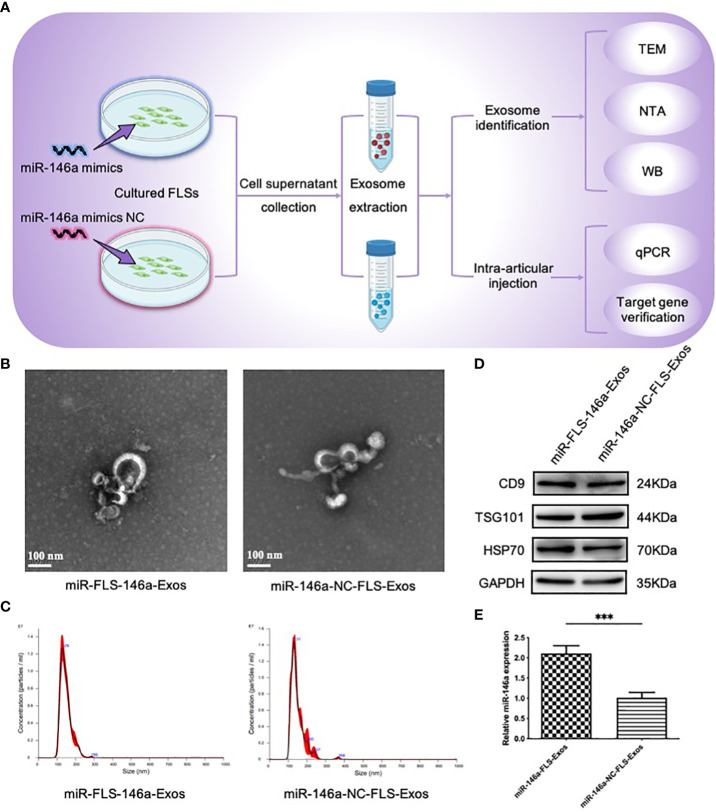
Extraction and identity assessment of exosome-like vesicles from FLSs pretreated with the miR-146a mimic. **(A)** FLSs were isolated and cultured in cell medium then transfected with the miR-146a mimic and miR-146a mimic NC. Exosome-like vesicles were extracted from the supernatant using supercentrifugation. **(B)** The morphology of exosome-like vesicles was observed using TEM (×100000). **(C)** The exosome particle size was measured using NTA (nm). **(D)** WB detected the exosome characteristic markers, CD9, TSG101, and HSP70. **(E)** qPCR was performed to detect the expression levels of miR-146a in exosome samples from the cell supernatant. Data are expressed as the mean ± SEM (n = 3). *** *p* < 0.0001.

The above *in vitro* experiments confirmed that exosomes were present in the synovium, and that synovial cells were likely to secrete exosomes. Next, FLSs from the synovial tissue were isolated and cultured, supernatants with miR-146a overexpression and a negative control were collected, exosomes were extracted separately, and their characteristics were evaluated. The TEM images showed elliptical or circular bodies. NTA showed that the particles had similar size distributions, with peak sizes of 129 and 131 nm. The WB results showed that these particles expressed the characteristic marker proteins of exosomes (CD9, TSG101, and HSP70). No significant differences in the morphology, size, or expression of marker proteins between the two groups was observed, which was consistent with the basic characteristics of exosomes. The expression levels of miR-146a in the exosome samples from the two groups were detected using qPCR; miR-146a-FLS-Exos levels were significantly higher than those of miR-146a-NC-FLS-Exos. These results confirm the role of miR-146a-FLS-Exos and the function of FLSs in exosome release (**Figure 4**).

**Figure 5:**
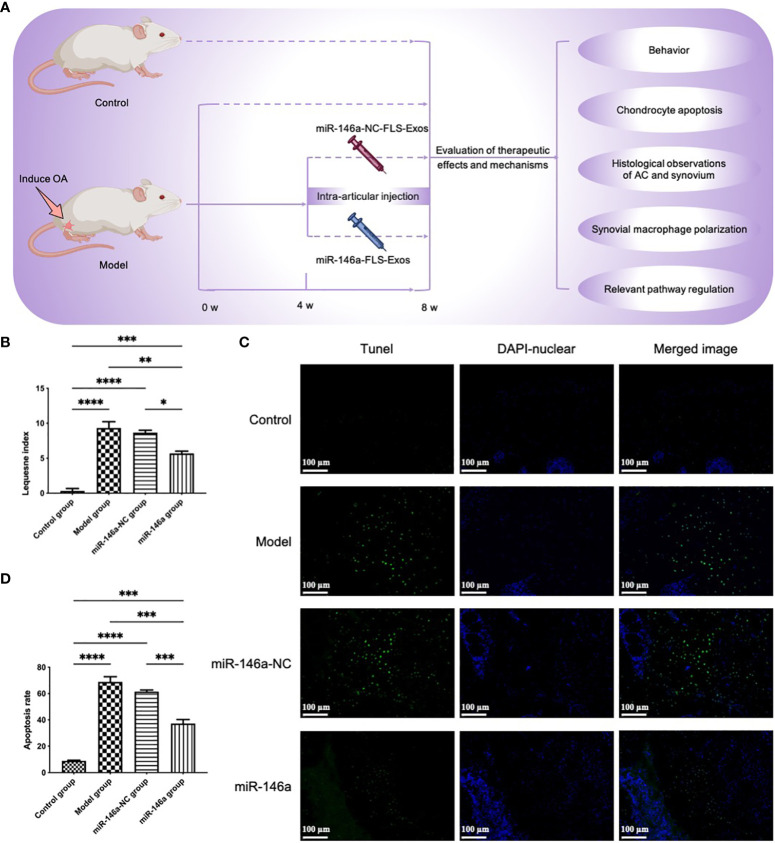
miR-146a-FLS-Exos improve behavior and inhibit apoptosis of OA chondrocytes *in vivo*. **(A)** Overview of the timeline of miR-146a-FLS-Exos treatment of OA. A rat model of OA was established using the modified Hulth method. The miR-146a-NC-FLS-Exos and miR-146a-FLS-Exos were injected into the injured joint from weeks 4–8 after surgery. The model and control groups without exosome treatment were used for comparison. The function of miR-146a-FLS-Exos in knee injuries of OA rats was evaluated using multiple assays. **(B)** The behavioral improvement of rats was evaluated using the Lequesne index. **(C, D)** Apoptosis in AC was analyzed using TUNEL staining and the proportion of apoptotic cells was assessed (×40). Data are expressed as the mean ± SEM (n = 3). **** *p* < 0.00001, *** *p* < 0.0001, ** *p* < 0.01, * *p* < 0.05.

To further evaluate whether exosomes extracted from FLSs under the intervention of miR-146a could play a more beneficial role in the cartilage of OA rats, miR-146a-FLS-Exos were injected into the injured joints of OA rats (**Figure 5A**). LG scores were first used to conduct a preliminary evaluation of the behavior of rats in each group (**Figure 5B**). In the model and miR-146a-NC groups, the behavioral scores of rats in the miR-146a group showed greater improvement, whereas there was no significant difference between the model and miR-146a-NC groups.

### 3.7 miR-146a-FLS-Exos inhibit the apoptosis of chondrocytes *in vivo*

The effect of FLS-miR-146a-Exos treatment on chondrocyte apoptosis and necrosis in the rat model of OA was evaluated using TUNEL staining. Compared with the small number of apoptotic cells in the control group, the positive rate of TUNEL in the AC of OA model rats was significantly higher, and the apoptotic cells showed specific green fluorescence staining throughout the layer of cartilage. The number and distribution of apoptotic cells in the miR-146a-NC group were similar to those observed in the OA group. The positivity rate of OA model rats treated with miR-146a-FLS-Exos was significantly reduced, and the chromatin fluorescence intensity was not as high as that in the above two groups. These results indicated that miR-146a-FLS-Exos could inhibit chondrocyte apoptosis to a certain extent *in vivo* (**Figures 5C, D**).

### 3.8 miR-146a-FLS-Exos inhibit cartilage degeneration and synovial proliferation *in vivo*

**Figure 6:**
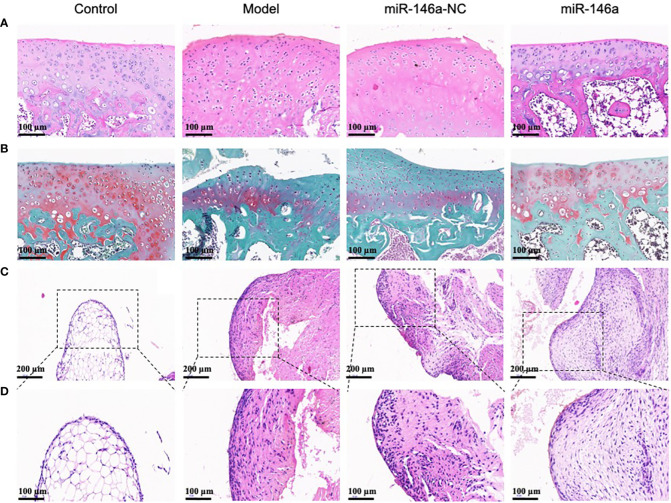
miR-146a-FLS-Exos inhibit cartilage degeneration and synovial inflammation in rats. **(A)** HE staining was used to observe the morphological characteristics of AC (×40). **(B)** Saffranin O-solid green staining was used to observe changes in the proteoglycan content in the cartilage matrix (×40). **(C, D)** HE staining was used to observe the morphological characteristics of the synovium (C. ×40, **(D)** ×20).

Histological observations of the AC using HE and saffron O-solid green staining are shown in **Figure 6**. The healthy AC of the control group had a smooth surface, uniform chondrocyte staining, regular cell stratification and morphology, a continuous and neat tide line, uniform matrix staining, and rich proteoglycan content with red staining. Compared with the model and miR-146a-NC groups, the AC in the miR-146a group showed a more complete chondrocyte arrangement on the cartilage surface and less matrix loss, such as proteoglycan, and the expression of miR-146a-FLS-Exos was similar to that which indicates a relatively mild degree of cartilage degeneration (**Figures 6A, B**).

HE staining of the synovial tissue also provided favorable histological evidence for the treatment of OA using miR-146a-FLS-Exos. The normal synovial lining layer consists of one or two layers of imbricated synoviocytes. The synovium in the model group was significantly thickened, the number of synoviocytes in the synovial lining layer was significantly increased, and the synoviocytes aggregated and were disordered. The synovium in the miR-146a-NC group was similar to that in the model group. Compared to the above two groups, the thickness of the synovium in the miR-146a group after treatment with miR-146a-FLS-Exos was significantly reduced, and the increase and aggregation of synoviocytes in the synovial lining layer were significantly relieved. These observations suggest that miR-146a-FLS-Exos can reduce synovial inflammation, which is consistent with its beneficial therapeutic effect on AC (**Figures 6C, D**).

### 3.9 miR-146a-FLS-Exos induced the polarization of synovial macrophages from M1 to M2

In addition to the large number of FLSs, which are the main forces of synovial fluid secretion, macrophages play an important role in synovial inflammation. The accumulation and phenotypic characteristics of macrophages in each group of synovial tissues were further identified using immunofluorescence. Consistent with the results of HE staining of the synovial tissue, high synovial hyperplasia and a large amount of cell infiltration were observed in the synovium of rats with OA. Compared with the control group, more F4/80 (macrophage marker)-positive fluorescence signals were detected in the model group, in which the number of iNOS (M1 macrophage marker)-positive cells increased substantially, whereas the proportion of CD206 (M2 macrophage marker)-positive cells decreased.

**Figure 7:**
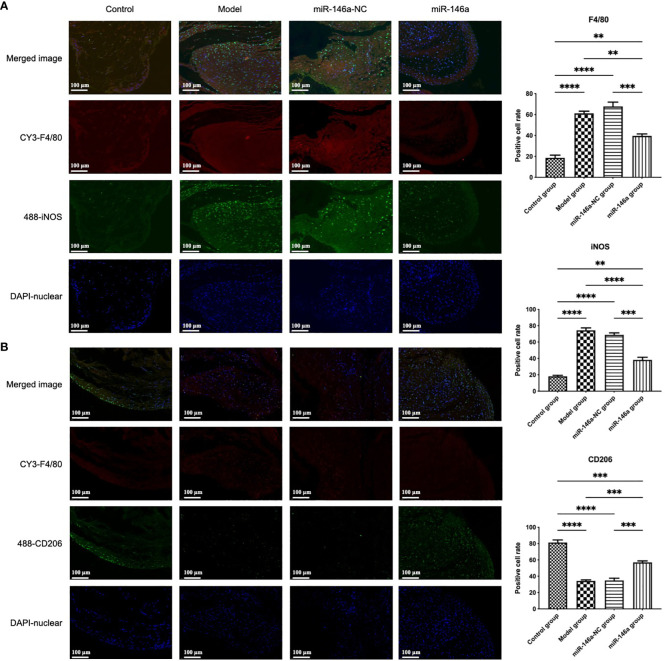
miR-146a-FLS-Exos induced polarization of synovial macrophages from M1 to M2. **(A)** Immunofluorescent co-localization of F4/80 and iNOS-positive macrophages in the rat synovium (×40). **(B)** Immunofluorescence co-localization of F4/80 and CD206-positive macrophage proportions in the rat synovium (×40). Data are expressed as the mean ± SEM (n = 3). **** *p* < 0.00001, *** *p* < 0.0001, ** *p* < 0.01.

These findings showed that there was more significant infiltration of synovial macrophages into the synovial membrane of rats in the model than in the control group, and there were more M1-polarized and fewer M2-polarized macrophages. Compared to the model and miR-146a-NC groups, the iNOS expression intensity of the miR-146a group was significantly decreased, whereas the CD206 expression intensity was significantly increased. These results indicate that FLS-Exos overexpressing miR-146a can effectively induce the polarization of synovial macrophages from the M1 phenotype to the M2 phenotype and have anti-inflammatory and tissue repair functions (**Figure 7**).

### 3.10 miR-146a-FLS-Exos inhibit the TLR4/TRAF6/NF-κB pathway *in vivo*

**Figure 8:**
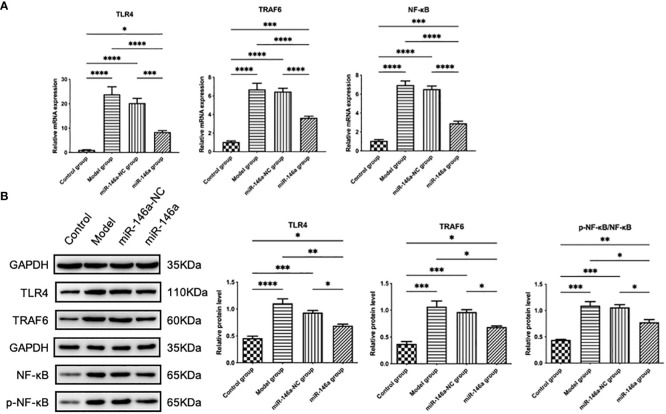
miR-146a-FLS-Exos regulate the TLR4/TRAF6/NF-κB pathway *in vivo*. **(A)** Relative mRNA expression levels of TLR4, TRAF6, and NF-κB were detected using qPCR. **(B)** The protein levels of TLR4, TRAF6, p-NF-κB and NF-κB were detected using WB. Data are expressed as the mean ± SEM (n = 3). **** *p* < 0.00001, *** *p* < 0.0001, ** *p* < 0.01, * *p* < 0.05.

The therapeutic effect of the miR-146a-FLS-Exos mentioned above may be related to the regulation of relevant pathways in the innate immune response, as confirmed by WB and qPCR results. The protein expression levels of TLR4, TRAF6, p-NF-κB, and NF-κB in rat AC were analyzed using WB, and the mRNA expression levels were detected using qPCR. The results showed that the protein and mRNA expression levels of TLR4, TRAF6, and NF-κB were consistent. The expression levels of the above genes in the model and miR-146a-NC groups were substantially higher than those in the control group, indicating that the TLR-mediated NF-κB pathway plays a role in the progression of OA. The expression level of the above genes in the AC of the miR-146a group was significantly inhibited compared with that of the model and miR-146a-NC groups, indicating that miR-146a-FLS-Exos can inhibit the TLR4/TRAF6/NF-κB pathway to a certain extent *in vivo* to achieve the therapeutic effect of slowing down the pathological process of OA (**Figure 8**).

## 4 Discussion

OA is a chronic degenerative disease that is caused by several factors. Lesions cover the entire joint, including major joint structures, such as the cartilage and synovium, as well as the liquid microenvironment composed of the synovial fluid in which they are located (22). Synovial fluid secreted by synoviocytes makes direct contact with joint tissues and provides conditions for material transport between cells (23). Synovial fluid contains a variety of bioactive substances and is a promising tool for the diagnosis, treatment, monitoring, and evaluation of OA (24–26). Exosomes in the synovial fluid, as nanoscale information material carriers with specific targeting, stability, and many other advantages, together with the inclusion of miRNAs and their mode of action, participate in the innate immune response and intercellular communication and are major contributors to the biology of OA (27, 28). In this study, the ability and mechanism of FLS-derived exosomal miRNAs to regulate OA pathology and degenerative knee changes were explored.

Currently, studies on exosome donor cells have focused on tumor cells and mesenchymal stem cells (MSCs) (29). The contents carried by exosomes are specific and mainly reflect the physiological and pathological changes in the source cells (30). Tumor-derived exosomes can carry tumor-specific antigens, miRNAs, proteins, and other substances that play a role in antitumor immunity (31). MSCs from the bone marrow, umbilical cord, and other tissues are the most commonly-used exosome-secreting cells owing to their differentiation potential, secretion, and immune regulatory functions (32). Our previous study found that in closed joint cavities, FLSs, the main cell type constituting the intimal structure of synovial tissue, can also secrete exosomes, such as MSCs and other donor cells, and exosomes carrying miRNA contents are released into the synovial fluid, affecting joint inflammation, cartilage destruction, and other pathological processes in OA (14). In this study, exosome samples consistent with the basic characteristics of exosomes were collected from the synovial fluid of OA model and healthy rats, preliminarily demonstrating the function of synoviocytes in exosome secretion.

Exosomes contain abundant miRNAs, and their stable phospholipid bilayer structure protects the integrity and stability of the miRNA content, preventing their degradation, dilution, capture, and clearance in various extracellular environments. As a newly-emerging gene that can independently affect the occurrence and development of OA, some studies have shown that the expression of miR-146a positively correlates with OA (33). Here, higher expression of miR-146a in the exosomes of the synovial fluid of OA rats than in healthy rats was detected, further suggesting that this exosomal miRNA is related to OA disease activity.

Exosomes release encapsulated miRNAs into recipient cells, alter the signaling process in these recipient cells, and achieve targeted intercellular communication (34). Therefore, it is very important to identify the specific signal transduction pathways associated with them, which can not only predict the risk of progression but also help to find more accurate treatments for OA. Data provided by GO and KEGG enrichment analysis have shown that miR-146a is involved in cellular senescence, the inflammatory response, and other activities, and its target gene is TRAF6. TRAF6, which is widely expressed in mammalian tissues and well-conserved in different species, is a key regulatory molecule of the NF-κB pathway in the innate immune response. It has been reported that the innate immune response is actively involved in the progression of OA in synovial inflammation and AC catabolic events. TLRs are important pattern recognition receptors in the innate immune system that recognize pathogens and other foreign microorganisms and play an important role in defense against various pathogens. TLR4, the earliest-discovered member of the TLR family, belongs to the type I transmembrane protein family. When activated, it can sense and recognize pathogen- and damage-associated molecular patterns during extracellular ligand recognition, collect transit proteins in intracellular signaling regions, activate the NF-κB signal transduction pathway, and induce the synthesis and release of a variety of cytokines and chemokines (35).

TargetScan online prediction software and a double-luciferase reporter assay further confirmed the relationship between miR-146a and TRAF6 expression. The expression of miR-146a was increased or inhibited in chondrocytes by transfection to evaluate the influence and mechanism of miR-146a. The results suggested that upregulation of miR-146a can alleviate apoptosis and inhibit the expression of TLR4, TRAF6, and NF-κB in chondrocytes *in vitro*. This may be because miR-146a can reduce the expression of TRAF6, a key adapter molecule in the TLR signaling cascade, affect the activation of the NF-κB pathway mediated by miR-146a, and reduce the activity of NF-κB. The NF-κB signaling pathway is an important regulatory pathway for apoptosis and has been shown to play a role in many cellular events (36).

In this study, we observed an initially paradoxical phenomenon: ample literature and previous research tended to affirm the inhibitory role of miR-146a on the TLR4/TRAF6/NF-κB inflammatory pathway (20), suggesting a potential protective role in the development of OA, and generally supporting the notion that the levels of miR-146a are elevated in individuals with OA compared to healthy individuals, consistent with our experimental results. This experimental data reveals an apparently contradictory phenomenon, which, however, is not contradictory in terms of pathological mechanisms but reflects a complex regulatory mechanism within the organism. Within this mechanism, the increase in miR-146a may be a regulatory response by cells to counteract inflammation and promote tissue homeostasis. Through precise molecular targeting, miR-146a inhibits the key inflammatory pathway of TLR4/TRAF6/NF-κB, thereby alleviating the inflammatory response at the molecular level and combating the progression of OA. Moreover, this phenomenon may reveal the dual role of miR-146a in the immune regulatory network, serving both as a direct modulator of the inflammatory response and as part of the feedback inhibition mechanism by which cells respond to disease stress (37). The transcription and subsequent maturation of miR-146a precursors induced by NF-κB exert further effects on signaling pathway nodes such as TLR4 and TRAF6, constituting an inherent regulatory circuit designed to reduce excessive activation of signal transduction, modulate immune cell differentiation, and inflammation mediators production (38). This discovery emphasizes the complexity of miR-146a as a potential therapeutic target and suggests that we must consider its multidimensional roles within the cellular signaling network when interpreting its expression patterns in autoimmune diseases such as OA. Therefore, our study not only reveals that there is no simple antagonistic relationship between the high expression of miR-146a and its protective role but indicates a multi-level, dynamic regulatory process, which may be an attempt by the organism to alleviate inflammation and restore tissue balance through its intrinsic molecular mechanisms. Future research needs to consider the role of miR-146a in different cell types and physiological contexts comprehensively to reveal its full role in disease regulation and ultimately use this knowledge to develop more precise therapeutic strategies.

Although miR-146a is an important immune response trimer in OA, its application is often greatly limited owing to problems such as easy degradation of free miRNAs, limited transmembrane ability, and possible immune response (39). Exosomes, as carriers of nucleic acids, can solve these problems. After confirming the beneficial effects of miR-146a overexpression on the targeted regulatory pathway of the innate immune system and chondrocyte apoptosis *in vitro*, the relevant role and mechanism of miR-146a-FLS-Exos in the treatment of modified Hulth-induced OA rats *in vivo* were evaluated. The injection of FLS-Exos pretreated with the miR-146a mimic to the joint cavity effectively promoted cartilage regeneration and prevented OA progression. OA-related pathological changes, including chondrocyte apoptosis, irregular cartilage surface morphology, chondrocyte stratification, tide line disorder, and proteoglycan loss, were substantially alleviated.

In addition to cartilage degradation, synovial inflammation is a common pathological feature of OA. Inflammatory cells proliferate, infiltrate, and even form pus, which thickens the synovial membrane and damages cartilage tissue, eventually leading to the progressive destruction of joint tissue and joint dysfunction. Although the synovial inflammatory response in OA is relatively mild compared to that in rheumatoid arthritis (RA), it is highly correlated with the pathological progression and pain manifestations of OA (40). In the synovial tissue, pathological changes in synovial cell hyperplasia, hypertrophy, disordered arrangement, fibrosis, and other synovial inflammation in rats treated with miR-146a-FLS-Exos were also uniformly and simultaneously alleviated along with cartilage injury. Similar to RA, the synovial cells in OA were mainly divided into a small number of type A synovial cells (macrophage-like synovial cells) and a large number of type B synovial cells (FLSs), as well as some inflammatory cells, such as monocytes, T cells, B cells, and plasma cells. In addition to a large number of FLSs that have been shown to secrete exosomes, macrophages in the synovial membrane, a major line of defense in the immune system, are activated during the pathological process of various diseases. Phenotypic changes in synovial macrophages reflect the extent and direction of the disease to a certain extent (41). This area of research requires further investigation. Activated synovial macrophages also play an important role in synovial inflammation (42, 43). However, there have been few studies on the mechanisms of macrophage activation in OA. The results of the present study suggest that articular injection of miR-146a-FLS-Exos can affect the phenotype of synovial macrophages, promote their polarization from the M1 to the M2 phenotype, and alleviate synovial inflammation.

**Figure 9:**
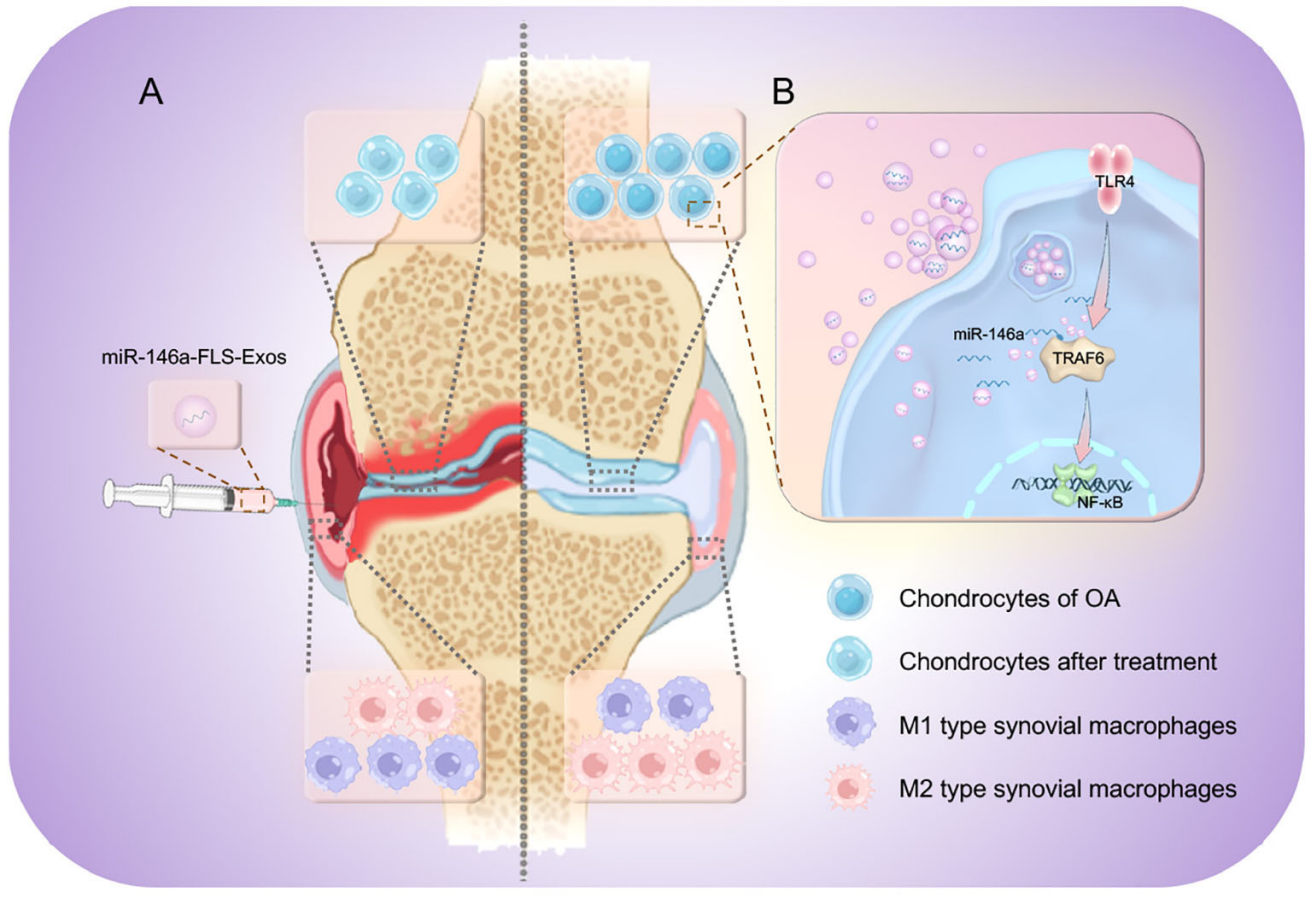
The therapeutic mechanism of miR-146a-FLS-Exos in OA. Synovial fibroblast-derived exosomes overexpressing miR-146a affected cartilage degradation and M1 polarization in macrophages. **(A)** miR-146a-FLS-Exos were injected into the articular cavity of OA rats to (1) reduce cartilage degeneration, (2) reduce synovial inflammation and proliferation, and induce synovial macrophage polarization from M1 to M2. **(B)** miR-146a-FLS-Exos can reduce the expression of TRAF6, a key adapter in the TLR4 receptor signaling cascade, and affect the activation of its mediated NF-κB pathway, thus, realizing the intercellular signaling process.

The above therapeutic effects are related to the targeted regulation of exosomal miR-146a on the TLR4/TRAF6/NF-κB pathway. Previously, miR-146a has mostly been reported in autoimmune diseases, such as RA. However, the present study confirmed the role of miR-146a in the pathological manifestations of OA through the exogenous intervention of exosomes with high expression of miR-146a. In terms of mechanisms, some studies have suggested that miR-146a can regulate diseases by participating in pathways related to innate immune responses. The current study confirmed that miR-146a can negatively regulate TRAF6, damage the activity of NF-κB, and inhibit the TLR4/TRAF6/NF-κB pathway (**Figure 9**).

However, it is unclear whether miR-146a has persistent changes in the maintenance of chronic diseases, such as OA, and whether it can influence changes in inflammatory responses, such as pain, by affecting molecular changes in the TLR4/TRAF6/NF-κB signaling pathway. Nevertheless, exosomes provide a key means of acquiring miRNA biomarkers and developing therapeutic intervention strategies for body fluids. In the current study, it was found that miR-146a-FLS-Exos may be novel biological factors in key OA processes, such as cartilage degradation and synovial macrophage polarization. In the future, new technologies and scientific methods may enable exosome miRNAs to be applied in the field of OA diagnosis and treatment and in public health strategies. The exploration of new substances to improve the timely diagnosis and effective management of OA has the potential to substantially improve the quality of life and health outcomes of patients with OA or those at risk of developing OA. This will reduce the global burden of OA.

## Data availability statement

The original contributions presented in the study are included in the article/supplementary material. Further inquiries can be directed to the corresponding authors.

## Ethics statement

The animal study was approved by the Animal Experiment Ethics Committee of China-Japan Friendship Hospital. The study was conducted in accordance with the local legislation and institutional requirements.

## Author contributions

HW: Writing – original draft. YZhang: Writing – review & editing. CZ: Writing – review & editing. YZhao: Writing – review & editing. JS: Writing – review & editing. XT: Writing – review & editing.
